# Intermittent hypoxia inhibits epinephrine-induced transcriptional changes in human aortic endothelial cells

**DOI:** 10.1038/s41598-022-21614-5

**Published:** 2022-10-13

**Authors:** Rengul Cetin-Atalay, Angelo Y. Meliton, Kaitlyn A. Sun, Mariel E. Glass, Parker S. Woods, Ying-Jie Peng, Yun Fang, Robert B. Hamanaka, Nanduri R. Prabhakar, Gökhan M. Mutlu

**Affiliations:** 1 Department of Medicine, University of Chicago, Chicago, IL USA; 2 Section of Pulmonary and Critical Care Medicine, University of Chicago, 5841 S. Maryland Avenue, MC6026, Chicago, IL 60637 USA; 3 Section of Emergency Medicine, University of Chicago, Chicago, IL USA; 4 Institute for Integrative Physiology, University of Chicago, Chicago, IL USA

**Keywords:** Experimental models of disease, Molecular medicine

## Abstract

Obstructive sleep apnea (OSA) is an independent risk factor for cardiovascular disease. While intermittent hypoxia (IH) and catecholamine release play an important role in this increased risk, the mechanisms are incompletely understood. We have recently reported that IH causes endothelial cell (EC) activation, an early phenomenon in the development of cardiovascular disease, via IH-induced catecholamine release. Here, we investigated the effects of IH and epinephrine on gene expression in human aortic ECs using RNA-sequencing. We found a significant overlap between IH and epinephrine-induced differentially expressed genes (DEGs) including enrichment in leukocyte migration, cytokine-cytokine receptor interaction, cell adhesion and angiogenesis. Epinephrine caused higher number of DEGs compared to IH. Interestingly, IH when combined with epinephrine had an inhibitory effect on epinephrine-induced gene expression. Combination of IH and epinephrine induced *MT1G* (Metallothionein 1G), which has been shown to be highly expressed in ECs from parts of aorta (i.e., aortic arch) where atherosclerosis is more likely to occur. In conclusion, epinephrine has a greater effect than IH on EC gene expression in terms of number of genes and their expression level. IH inhibited the epinephrine-induced transcriptional response. Further investigation of the interaction between IH and epinephrine is needed to better understand how OSA causes cardiovascular disease.

## Introduction

Obstructive sleep apnea (OSA) is a highly prevalent sleep disorder in the adult population. The prevalence of OSA has been estimated to be up to 34% of adult men and 17% in adult women^1,2^. A recent analysis of global data estimated that close of 1 billion adults between the ages of 30 and 69 have OSA of which approximately half have moderate to severe OSA^3^. OSA is an independent risk factor for cardiovascular disease including hypertension, stroke, heart failure, coronary artery disease and atrial fibrillation^4–6^. Patients with cardiovascular disease have high prevalence of OSA (40–60%)^7^. In addition, severe OSA (apnea hypopnea index equal or greater than 30) is associated with increased cardiovascular and all-cause mortality^8^. However, the mechanisms by which OSA causes cardiovascular disease are not completely understood.

The pathophysiology of OSA includes intermittent hypoxia (IH), intermittent hypercapnia, intrathoracic pressure swings, arousals, and sleep fragmentation^9–12^. IH is a hallmark manifestation of OSA^10,13^. The severity of hypoxia or the hypoxia burden has recently been shown to be a strong predictor of cardiovascular and all-cause mortality in patients with OSA^14^. IH episodes lead to increased sympathetic tone and elevation of circulating and urinary catecholamine levels^9,10,15–19^. IH may thus mediate its effects either directly through the effects of hypoxia, or indirectly via catecholamines.

Endothelial cells (ECs) play an important role in the pathogenesis of cardiovascular disease. Activation of ECs characterized by the expression of pro-inflammatory cytokines and adhesion molecules, is an early process in the development of cardiovascular disease^20–24^. OSA may affect EC function as they are directly exposed to the changes in oxygen concentration as well as to circulating catecholamines. In fact, OSA patients exhibit markers of EC activation, which are reversed by CPAP treatment^20–22,25–27^.

Since EC activation or dysfunction may precede the development of cardiovascular disease, there has been significant interest into determining mechanisms by which OSA affects EC function. Studies evaluating the effects of IH on ECs have shown increased production of pro-inflammatory cytokines, and adhesion molecules consistent with EC activation^28–33^. Using gene array, Polotsky et al. found that short-term (8 h) exposure to human aortic endothelial cells (HAECs) led to increased expression of pro-inflammatory cytokines particularly IL-8 and NRF2 target genes; however, IH changed neither hypoxia-inducible factor-1α (*HIF1A*) nor HIF target gene expression^28^. Similar to HAECs, murine lung ECs also increased the expression of cytokines in response to IH^29^. Interestingly, in this case, IH reduced the expression of genes involved in the management of oxidative stress. While short-term (4–24 h) exposure to IH was well-tolerated, longer exposures to IH led to cell death. Exposure of human umbilical vein ECs (HUVECs) to IH induced the expression of adhesion molecules and endothelial-cell-specific molecule-1^30^. In contrast to Polotsky et al., IH-induced changes were found to be HIF-dependent. Other studies using HUVECs^31^ and HAECs^32^ showed increased permeability evidenced by decreased transendothelial electrical resistance and increased expression of vascular endothelial cadherin (VE-cadherin). IH-induced increased permeability in HAECs was both HIF and oxidant dependent^32^.

In contrast to these studies showing that IH may directly cause EC activation, we have recently shown that EC activation occurs indirectly via catecholamines in a murine model of OSA^34^. We found that EC activation in mice subjected to IH mimicking changes in blood O_2_ saturation in OSA was abolished when catecholamine release was inhibited by carotid body ablation, adrenalectomy or by chemical sympathectomy and thus, was not due to direct effect of IH, but rather indirectly through IH-induced sympathetic activation-derived epinephrine^34^. While our recent findings provide a novel mechanistic role for IH-induced sympathetic activation in EC activation, our previous study analyzed a limited number of genes involved in inflammation and immune cell adhesion that were implicated in EC activation. Building on our recent findings, in this study we sought to determine the gene expression changes in ECs induced by IH and epinephrine alone or together using an unbiased approach with RNA-seq, and to test the hypothesis that epinephrine modulates the effect of IH-induced gene expression changes in ECs.

## Results

### Exposure to IH induces genes that are involved in leukocyte migration, angiogenesis, and extracellular matrix organization

We have recently shown that exposure of mice to IH to model blood O_2_ desaturations encountered during OSA activates ECs as evidenced by increased expression of pro-inflammatory cytokines and adhesion molecules^34^. Moreover, our studies using clinically relevant hypoxia levels showed that IH does not cause EC activation directly but indirectly via sympathetic nervous system activation and catecholamine release. We sought to expand this initial study by using RNA-sequencing to determine the effects of IH and epinephrine on EC gene expression in an unbiased manner. We first exposed human aortic endothelial cells (HAECs) to normoxia (20% O_2_) or IH (5% O_2_) for 60 cycles and collected RNA for sequencing. Control cells were exposed to normoxia (20% O_2_) contemporaneously. We did not observe any morphological changes in EC exposed to IH compared to control cells (Supplementary Figure S1). We measured O_2_ concentrations in media in which the cells were intermittently exposed. At 37 °C, the dissolved O_2_ content in normoxia media ranged from 6.2 to 6.7 mg/l (99.18–99.58% air (20% O_2_)-saturated). In the hypoxia media, the dissolved O_2_ content ranged from 1.4 to 1.7 mg/l (20.81–25.27% hypoxia (5% O_2_-saturated).

**Figure 1:**
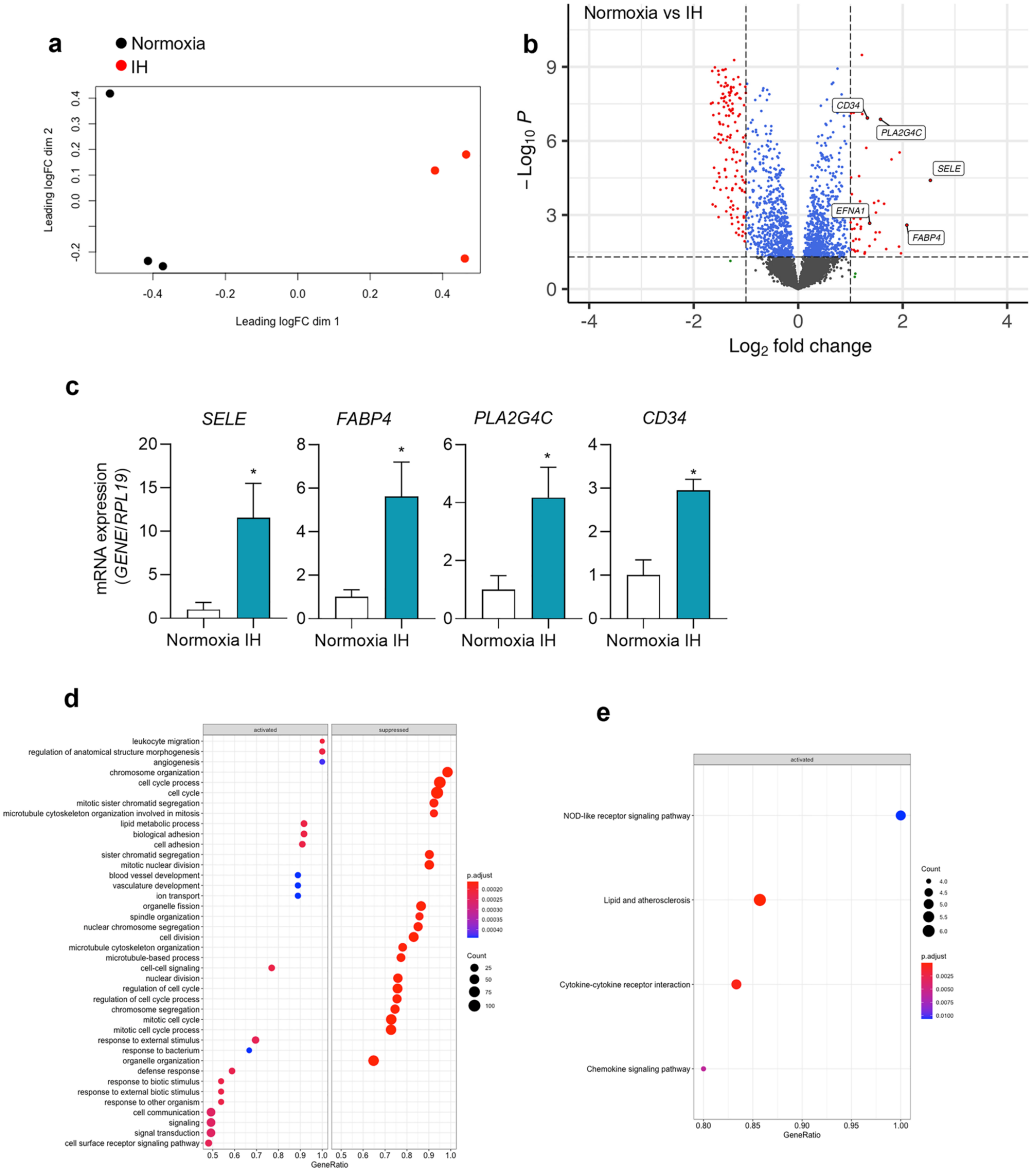
Transcriptional changes in human aortic endothelial cells after exposure to intermittent hypoxia. Human Aortic Endothelial Cells (HAECs) were exposed to 60 cycles of IH (5%-20% O_2_) or normoxia (20% O_2_) (control). RNA-sequencing was performed from RNA isolated from 3 biological replicates. (**a**) Multidimensional scaling plot of differentially expressed genes in HAECs under IH compared to control normoxia. LogFC dim 1, and dim 2: base 2 logarithm of fold change dimension 1 and 2. (**b**) Volcano plot of -Log10 (*p* values) vs. Log2 fold change for DEGs and top significantly upregulated genes in IH. The -log10 (*p* values) represents the level of significance of each gene while log2 fold change represents the difference between the levels of expression for each gene. (**c**) Using qPCR, we measured mRNA expression of E-selectin (*SELE*), Fatty acid binding protein 4 (*FABP4*), Cytosolic phospholipase A2 gamma (*PLA2G4C*), and Hematopoietic progenitor cell antigen CD34 (*CD34*) in HAECs under control normoxia and IH (data are shown as mean ± SD, *n* = 6, biologic replicates). *p < 0.05. Parameters for DEG significance were set to absolute fold change ≥ 2 and FDR adjusted *p* value ≤ 0.05. Activated and suppressed (**d**) gene ontology biological processes (GO BP) and (**e**) activated KEGG pathways were identified by gene set enrichment analysis of DEGs in HAECs under IH compared to normoxia. The raw source data for RNA-seq are accessible via GEO (GSE205050). https://www.ncbi.nlm.nih.gov/geo/query/acc.cgi?acc=GSE205050.

Multi-dimensional scaling (MDS) plot showed linearly separable groups of gene expressions between normoxia- and IH-exposed ECs (Fig. 1a). Analysis of RNA-seq data showed 184 differentially expressed genes (DEGs) between normoxia- and IH-exposed HAECs. Compared to normoxia control, exposure to IH caused upregulation of 50 genes and downregulated 134 genes (Fig. 1b). *HIF1A* was not included among the DEGs under IH. We confirmed that IH did not induce HIF-1α protein or gene expression (Supplementary Figure S2). IH also failed to induce HIF target genes (VEGFA, EGLN3) (Supplementary Figure S2). *SELE*, which encodes E-selectin was identified as the top upregulated gene by IH (Fig. 1b). E-selectin is a cell adhesion molecule that is expressed only on endothelial cells in response to cytokines^35^. *SELE* is involved in the adhesion of neutrophils to the cytokine-activated endothelium^36^. Other top upregulated genes included Fatty acid binding protein 4 (*FABP4*), Cytosolic phospholipase A2 gamma (*PLA2G4C*), Ephrin A1 (*EFNA1*), and Hematopoietic progenitor cell antigen CD34 (*CD34*). FABP4 plays an important role in angiogenesis. Increased expression causes endothelial dysfunction and is associated with cardiovascular disease^37^. EFNA1 was initially discovered as a TNF-induced protein in human umbilical vein endothelial cells and increases adhesion and migration of ECs^38^. CD34 is an important adhesion molecule that regulates immune cell migration. Using qPCR, we confirmed the IH-induced increase in the expression of these genes except for *EFNA1* (Fig. 1c).

To better interpret the gene expression data, we performed gene ontology biological process (GO BP) enrichment analysis (Fig. 1d) and KEGG pathway enrichment analysis (Fig. 1e). GO BP enrichment analysis showed that leukocyte migration, extracellular matrix organization, cell adhesion and blood vessel development biological processes were activated under IH (Fig. 1d). In contrast, IH suppressed cell division, microtubule based process, and regulation of cell cycle biological processes. KEGG pathway enrichment analysis (Fig. 1e) showed that NOD-like receptor signaling pathway, lipid and atherosclerosis, cytokine-cytokine receptor interaction and chemokine signaling pathways were activated under IH. These results suggest that exposure of ECs to IH alone can alter the expression of genes enriched in pathways that have been previously shown to be associated with the development of cardiovascular disease.

### Epinephrine also induces genes that are involved in leukocyte migration, angiogenesis, and matrix organization

**Figure 2:**
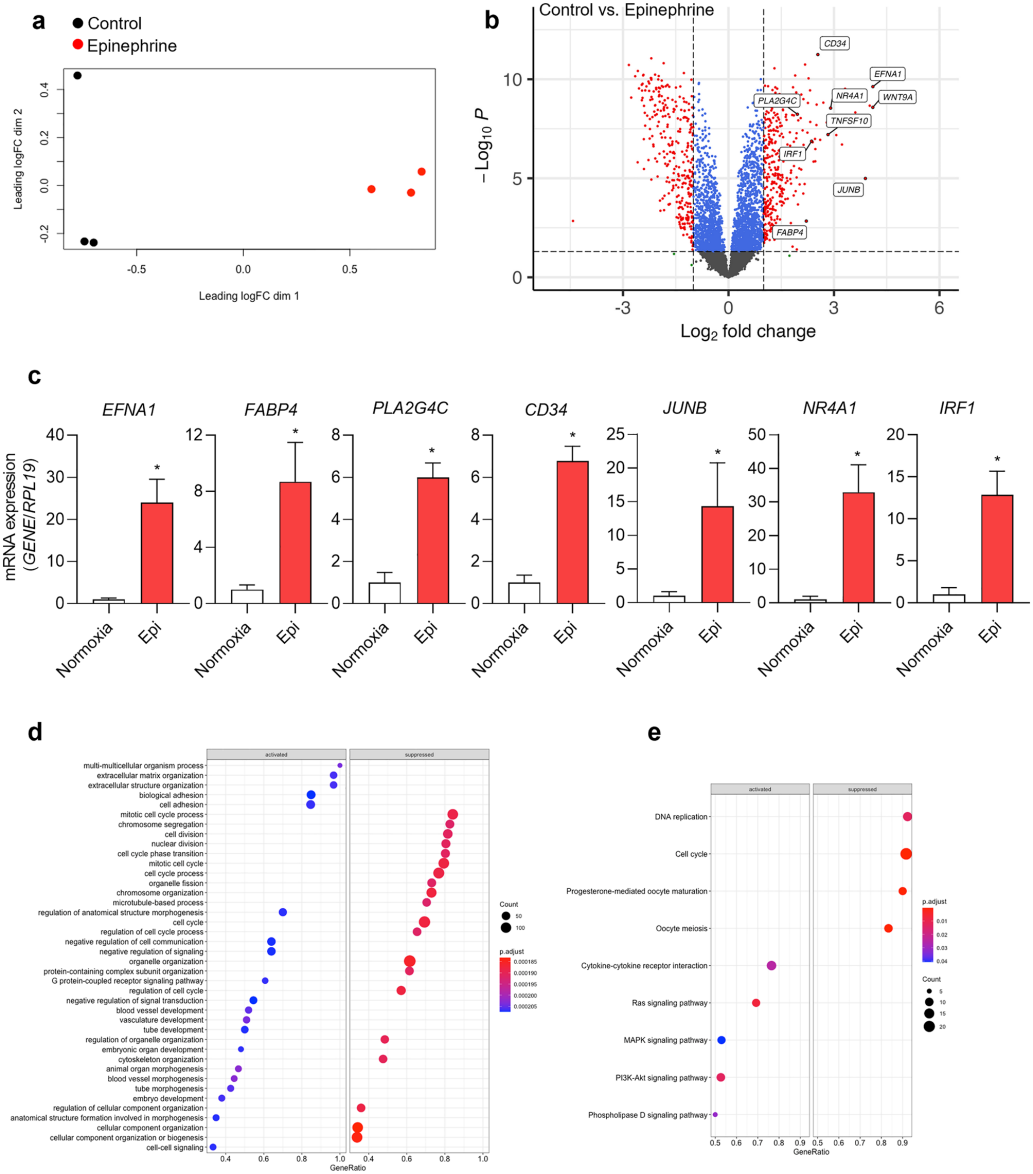
Transcriptional changes in human aortic endothelial cells after treatment with epinephrine. HAECs were treated with epinephrine (10 µM) or vehicle control for 7 h under normoxia (20% O_2_) using the perfusion system. RNA-sequencing was performed from RNA isolated 3 biological replicates. (**a**) Multidimensional scaling plot of differentially expressed genes in HAECs treated with epinephrine or vehicle control. LogFC dim 1, and dim 2: base 2 logarithm of fold change dimension 1 and 2. (**b**) Volcano plot of -Log10 (*p* values) vs. Log2 fold change for DEGs and top significantly upregulated genes upon epinephrine treatment. The -log10 (*p* values) represents the level of significance of each gene while log2 fold change represents the difference between the levels of expression for each gene. (**c**) Using qPCR, we measured mRNA expression of *EFNA1*, *FABP4*, *PLA2G4C*, *CD34*, Transcription factor jun-B (*JUNB*), Nuclear receptor subfamily 4 group A member 1 (*NR4A1*) and Interferon regulatory factor 1 (*IRF1*) in HAECs under control normoxia and IH (data are shown as mean ± SD, *n* = 6, biologic replicates). *p < 0.05. Parameters for DEG significance were set to absolute fold change ≥ 2 and FDR adjusted *p* value ≤ 0.05. Activated and suppressed gene (**d**) ontology biological processes (GO BP) and (**e**) activated and suppressed KEGG pathways were identified by gene set enrichment analysis of DEGs in HAECs treated with epinephrine compared to control treatment.

To determine the effect of epinephrine on EC gene expression, we treated HAECs cultured under normoxia with epinephrine (10 µM) or control for 7 h, which corresponds to the time of 60 cycles of IH and collected RNA for sequencing. MDS plot clearly showed separate gene expressions clusters of control and epinephrine treatment (Fig. 2a). Analysis of RNA-seq data showed that compared to IH alone, epinephrine caused a higher number of significant DEGs. There were 635 DEGs between vehicle- and epinephrine-treated HAECs. Compared to control treatment, epinephrine treatment upregulated the expression of 329 genes and downregulated 306 genes.

*EFNA1* was the top upregulated gene by epinephrine (Fig. 2b). In addition to *EFNA1*, which was also upregulated by IH, epinephrine induced the expression of genes that were also upregulated by IH such as *FABP4*, *PLA2G4C*, *EFNA1*, and *CD34* (Figs. 1b, 2b). Top genes upregulated by epinephrine included *JUNB*, *NR4A1* and *IRF1*. *JUNB* (Transcription factor jun-B) is required for endothelial morphogenesis^39^. *NR4A1* (Nuclear receptor subfamily 4 group A member 1) is expressed highly in aortic ECs in response to oxidized low-density lipoprotein in vitro or isolated from high-fat treated mice *in vivo*^40^. It plays an important role in atherosclerosis formation. *IRF1* (Interferon regulatory factor 1) is induced in ECs by inflammatory stimuli such as LPS and regulates VCAM-1 expression independent of NF-κB, but not E-selectin expression^41^. Genes that were upregulated by epinephrine were validated by qPCR (Fig. 2c).

To better interpret the comparative gene expression data, we performed GO BP enrichment analysis (Fig. 2d) and KEGG pathway enrichment analysis with the significant DEG set (Fig. 2e). GO BP enrichment analysis showed that extracellular matrix organization, cell adhesion, leukocyte migration, and blood vessel development biological processes were activated in response to epinephrine treatment (Fig. 2d). In contrast, epinephrine suppressed DNA repair, organelle organization, and cell cycle biological processes. KEGG pathway enrichment analysis (Fig. 2e) showed that cytokine-cytokine receptor interaction, Ras signaling, MAPK signaling, PI3K-Akt signaling, and Phospholipase D signaling pathways were activated in response to epinephrine. Epinephrine suppressed DNA replication and cell cycle pathways. These results suggest that similar to IH alone, epinephrine can independently alter the expression of genes enriched in pathways that have been previously shown to be associated with the development of cardiovascular disease.

### Epinephrine induces significantly higher number of DEGs compared to IH in endothelial cells

**Figure 3:**
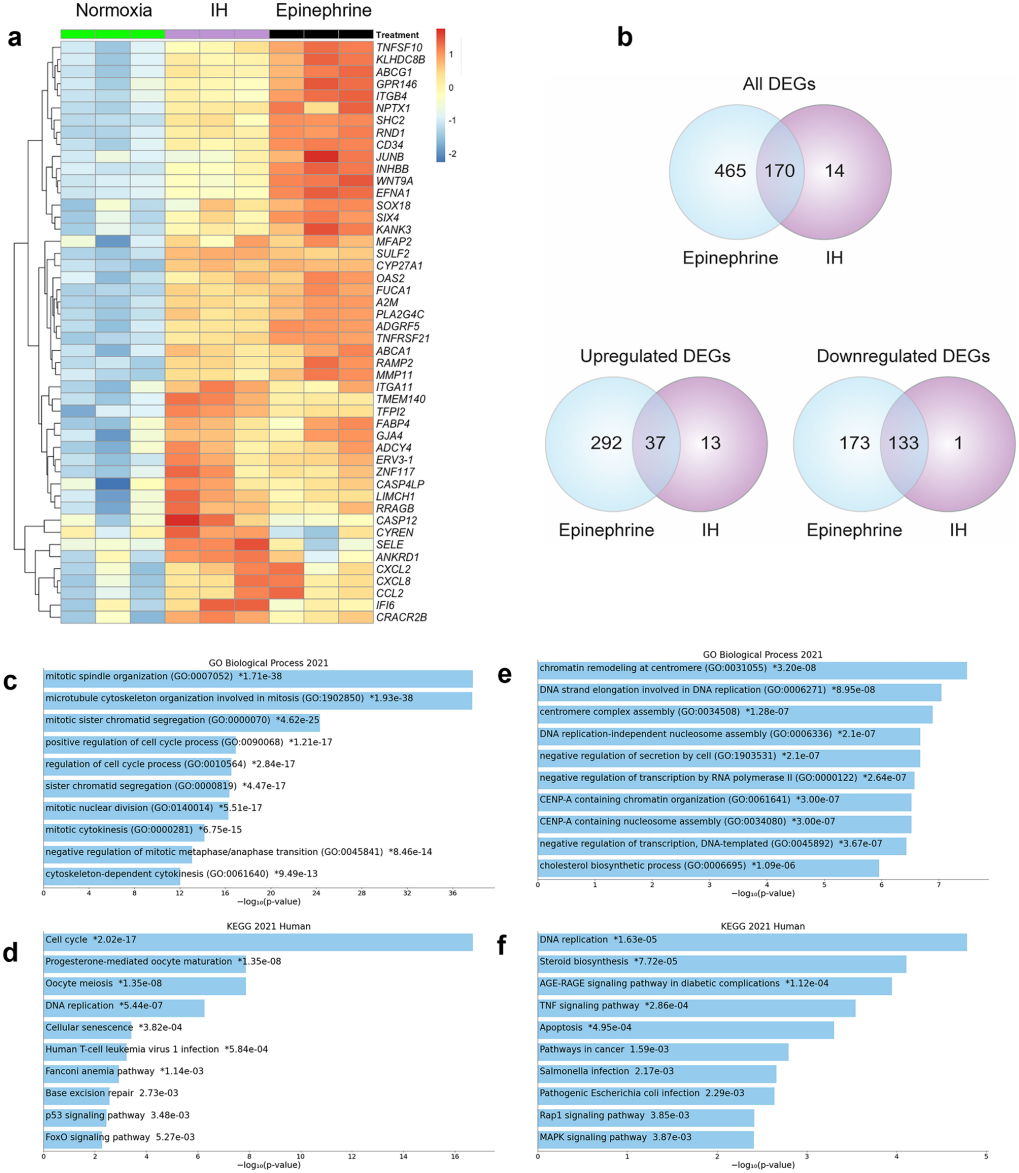
Epinephrine induces significantly higher number of DEGs compared to IH in human aortic endothelial cells. Differentially expressed gene profiles of HAECs, which were treated with epinephrine (10 µM) under normoxia or were exposed to 60 cycles of IH (5%-20% O_2_) perfusion system, were comparatively analyzed. (**a**) Heatmap of top significantly upregulated genes by IH in normoxia controls and IH- or epinephrine-treated HAECs. Z-score normalized expression values are shown in heat map, scale bar represents logFC values. “Pretty heatmaps R pheatmap package” from Z-score normalized expression values. https://cran.r-project.org/web/packages/pheatmap/pheatmap.pdf (**b**) Venn diagrams show differentially expressed genes (absolute fold change ≥ 2 and *p* value ≤ 0.05) in epinephrine-treated normoxic ECs (635 DEGs), and IH-exposed HAECs (184 DEGs). GO biological process (**c**) and KEGG pathway (**d**) enrichment analysis of gene set that were common between IH and epinephrine by Enrichr gene set search engine. GO biological process (**e**) and KEGG pathway (**f**) enrichment analysis of gene set that were specific to epinephrine by Enrichr gene set search engine.

Compared to IH, epinephrine caused a significantly higher number of DEGs in ECs (635 vs. 184). Furthermore, comparison of the expression levels of top significantly upregulated genes by IH showed that the extent of upregulation of the majority of genes were higher in epinephrine treated cells compared to IH (Fig. 3a). Despite the differences in the number of DEGs, GO BP enrichment analysis surprisingly showed activation of similar biological processes. Both IH and epinephrine induced extracellular matrix organization, cell adhesion, leukocyte migration, and blood vessel development. However, KEGG pathway analysis showed pathway differences. Both IH and epinephrine activated cytokine-cytokine receptor activation. IH activated NOD-like receptor signaling and lipid and atherosclerosis, whereas epinephrine induced Ras, MAPK, PI3K-Akt, and Phospholipase D signaling (Figs. 1e, 2e).

To better understand the similarities and differences between IH and epinephrine on EC gene expression, we compared the DEGs between two treatments (Fig. 3b). The majority of DEGs in response to IH were also observed in response to epinephrine treatment (170 out of 184 genes, 92%). Only 14 genes were specific to IH, whereas 465 genes were specific to epinephrine. DEGs specific to IH included *SELE, CASP12, RRAGB, TUBB4B, LIMCH1, IFI6, ANKRD1, CRACR2B, TNFAIP8L3, TMEM140, CASP4LP, SMARCD3, CYREN* and *TFPI2*. Due to low gene count, we could not perform GO BP or KEGG pathway enrichment analysis on the gene set that was significantly altered only under IH.

Enrichr enrichment analysis search engine tool for mammalian gene sets was used for the common 170 significant DEGs between IH and epinephrine. Analysis of GO BP for this gene set showed that microtubule cytoskeleton organization involved in mitosis, mitotic spindle elongation, and midzone assembly were the top processes that were common for IH and epinephrine (Fig. 3c). KEGG pathway analysis showed cell cycle, DNA replication, and cellular senescence among the top pathways (Fig. 3d). GO biological process analysis of the gene set (465 genes) that was specific to epinephrine showed chromatin remodeling at centromere, DNA strand elongation involved in DNA replication, as well as cholesterol biosynthetic processes (Fig. 3e). KEGG pathway analysis demonstrated the gene set was enriched in DNA replication, steroid biosynthesis, ACE-RAGE signaling, TNF signaling, and pathogenic E Coli infection (Fig. 3f). These results suggested that exposure to IH and epinephrine share a significant number of DEGs enriched in mitosis and microtubule organization, cell cycle and DNA replication while epinephrine related DEGs are enriched further in DNA replication and chromatin remodeling. In addition, genes that are specific to epinephrine treatment are enriched in metabolic and inflammatory pathways.

### Combining IH with epinephrine significantly alters epinephrine-induced changes in gene expression

Since epinephrine release occurs during IH^34^, we then sought to determine the effect of IH and epinephrine together on gene expression in ECs. To do this, we exposed HAECs to 60 cycles of IH in the presence or absence of epinephrine and isolated RNA for sequencing. We then compared the effects of IH on epinephrine-related DEGs and epinephrine on IH-related DEGs.

**Figure 4:**
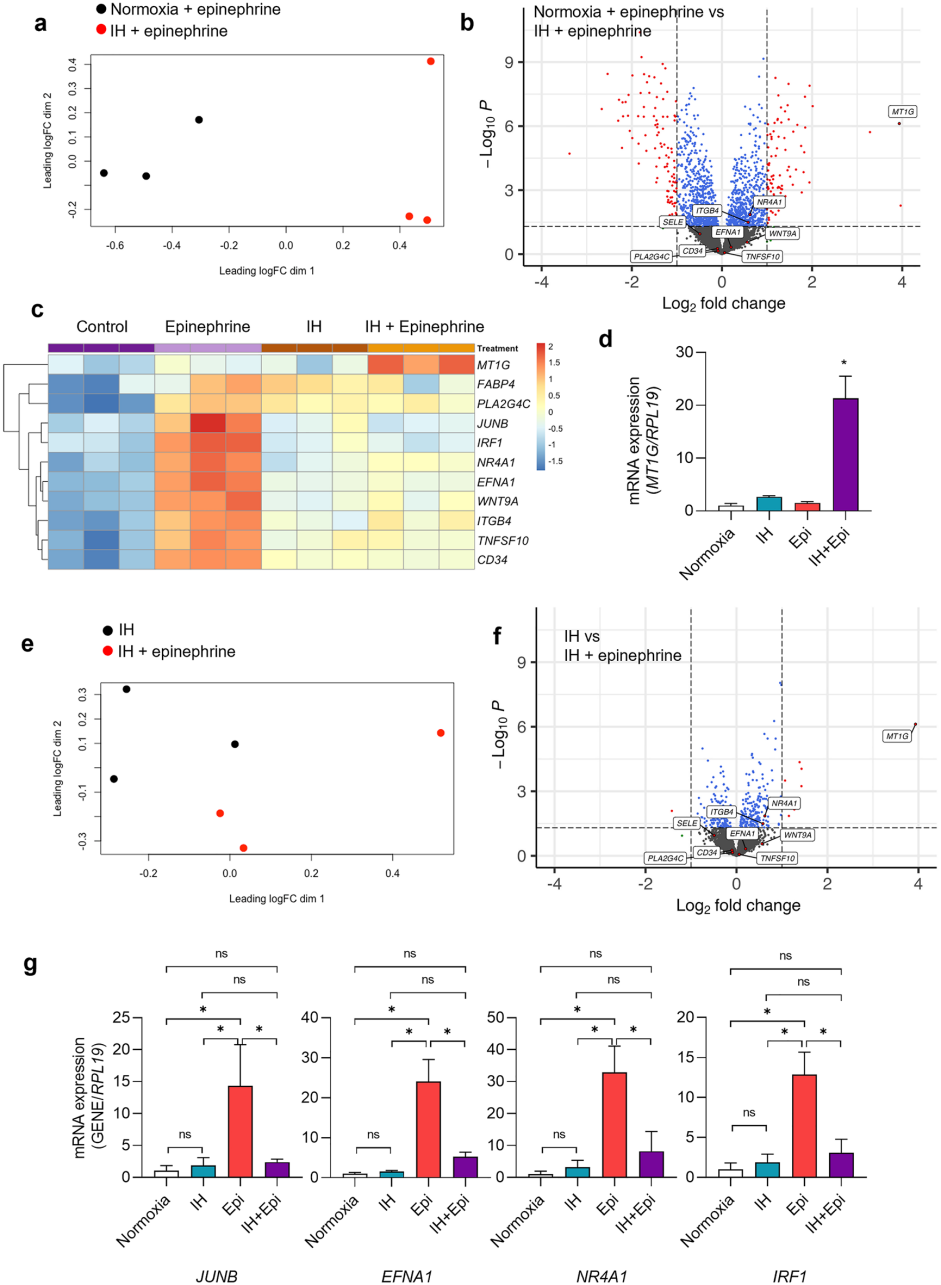
Combining IH with epinephrine significantly alters epinephrine-induced changes in gene expression. HAECs were exposed to 60 cycles of IH (5%-20% O_2_) in the presence of 10 µM epinephrine and vehicle control. We then isolated RNA from 3 biologic replicates and performed RNA-sequencing. (**a**) Multidimensional scaling plot and (**b**) Volcano plots of DEGs in HAECs treated with normoxia + epinephrine and IH + epinephrine. (**c**) Heat map displaying the expression level of top epinephrine-upregulated genes in HAECs treated with epinephrine or vehicle control under normoxic and IH conditions. “Pretty heatmaps R package pheatmap package” from Z-score normalized expression values. https://cran.r-project.org/web/packages/pheatmap/pheatmap.pdf (**d**) Using qPCR, we measured mRNA expression of *MT1G* measured by qPCR in HAECs treated and untreated with 10 µm epinephrine under IH and normoxia (mean ± SD, *n* = 6, biologic replicates). *p < 0.05. (**e**) Multidimensional scaling plot of DEGs in HAECs treated with IH in the presence or absence of epinephrine (10 µM). (**f**) Volcano plot of DEGs in HAECs treated with IH in the presence or absence of epinephrine. Metallothionein-1G (*MT1G)* is highly differentially upregulated under IH supplemented with 10 µM epinephrine. (**g**) We also measured mRNA expression of epinephrine-upregulated genes, *JUNB, EFNA1*, *IRF1*, and *NR4A1* in HAECs treated with epinephrine or vehicle control under normoxic and IH conditions (mean ± SD, *n* = 6, biologic replicates). *p < 0.05.

To evaluate the effect of IH on epinephrine-related DEGs, we analyzed gene expression between normoxia- and epinephrine-treated cells or IH + epinephrine-treated cells. MDS plot showed linearly separable clustering of normoxia + epinephrine and IH + epinephrine samples (Fig. 4a). There were 184 DEGs between two groups. Compared to normoxia + epinephrine treatment, IH + epinephrine upregulated 85 genes and downregulated 99 genes.

The most significantly upregulated gene by the addition IH to epinephrine treatment was metallothionein-1G (*MT1G)*, which is a member of low- molecular-weight cysteine-rich metallothionein protein family, and is involved in zinc and redox metabolism, and displays immunomodulatory functions in inflammatory diseases^42^ (Fig. 4b–d). We confirmed the significantly higher mRNA expression of *MT1G* by qPCR upon epinephrine treatment under IH in HAECs (Fig. 4d). Although neither IH nor epinephrine alone affected the expression of this gene, combination of IH with epinephrine induced it. Interestingly, we found that addition of IH to epinephrine reduced some of the top upregulated genes under epinephrine alone, including *EFNA1, JUNB, NRARP, IRF1*, and *NR4A1* (Fig. 4c), which was confirmed by qPCR (Fig. 4g). In contrast, addition of IH also increased some of the highly downregulated genes under epinephrine including AHR, and KITLG. *AHR* (Aryl hydrocarbon receptor) is highly expressed in endothelial cells. Its expression has been linked to blood pressure regulation and aging related changes in vasculature^43^. *KITLG* (KIT Ligand), also known as stem cell factor, is regulated by VEGF receptor blockade^44^. Collectively, IH reduced the effect of epinephrine on EC gene expression.

We next analyzed the gene expression between IH and IH + epinephrine. Clustering of IH samples and IH + epinephrine samples was less clear. (Fig. 4e). There were 11 DEGs between two groups. *MT1G, ADAMTS4, DKK1, CLCG1, NPTX1, GPRC5A, DUSP4* and *IL6* were upregulated and *TNFAIP8L3* was the only gene that was downregulated by IH (Fig. 4f). These results suggested that combination of IH and epinephrine do not provide additional differentially gene expression compared to IH alone.

## Discussion

OSA is a common disorder affecting up to 20% of the adult population in the US and close to 1 billion people worldwide. In addition to its consequences on cognitive function, particularly excessive daytime sleepiness and memory problems, OSA is significantly associated with cardiovascular disease. Our understanding of how OSA exerts its biologic effects on the cardiovascular system is not completely understood. As one of the hallmarks of OSA, IH plays a key role in the pathogenesis of OSA-related cardiovascular disease given that the severity of hypoxia predicts cardiovascular and all-cause mortality in patients with OSA^14^.

Endothelial cells are important in cardiovascular homeostasis, including regulation of blood pressure. It is not surprising that EC dysfunction and activation play a critical role in the development of cardiovascular disease and precedes clinically overt disease^20–24^. OSA is associated with markers of EC dysfunction and activation^45^. Exposure to low level continuous hypoxia or IH upregulates proinflammatory and adhesion genes which are reported to be responsible of EC activation^28,30,33^. Murine models of OSA usually focus only on IH as the factor to regulating EC function^29^. We have recently shown that exposure to IH induces EC activation not directly, but indirectly via epinephrine release in response to IH-induced carotid body sensing and sympathetic activation^34^. In this study, we sought to expand our initial studies to better understand the effects of IH and epinephrine on EC function by determining the gene expression profiles in response to IH or epinephrine alone, or in combination. We found that epinephrine induced a significantly higher number of differentially expressed genes compared to IH alone. Furthermore, the expression levels of the top genes induced by IH were significantly lower compared to the expression levels induced by epinephrine. Surprisingly, the majority (92%) of significant DEGs we identified under IH were also seen in response to epinephrine. Analysis of Gene Ontology Biologic Processes (GO_BPs) showed that the genes that were common between IH and epinephrine were enriched in leukocyte migration, extracellular matrix organization, cell adhesion and blood vessel development. Using KEGG pathway enrichment analysis we identified several pathways specific for epinephrine, including Ras signaling, MAPK signaling, PI3K-Akt signaling, and Phospholipase D signaling pathways in addition to cytokine-cytokine receptor interaction. Collectively, these results suggested that (1) epinephrine has a greater impact than IH on gene expression levels and profiles, (2) IH- and epinephrine-induced gene expression profiles are similar in terms of enrichment of biological processes (GO_BP), and (3) epinephrine has a greater effect than IH on gene expression associated with signaling pathways as assessed by KEGG pathway enrichment analysis.

We found that IH or epinephrine alone regulates leukocyte migration, angiogenesis, and cell matrix organization (GO_BP and KEGG) pathways in HAECs. These pathways are known to be regulated by various pathophysiologic changes such as oxidative stress, chronic inflammation, and sympathetic activation that occur in patients with OSA, and animals exposed to IH to model OSA^32^. Importantly, these pathways and molecular events are responsible for EC dysfunction, hypertension, and atherosclerosis in OSA. Both patients with OSA and animals exposed to IH have elevated oxidative stress with higher production of inflammatory cytokines and adhesion molecules and antioxidant agents have been shown to improve oxidative stress-associated endothelial dysfunction^31,32^.

Pathways we identified following IH are similar to the data from previous studies which evaluated the effect of IH on EC function showing upregulation of cytokines, adhesion molecules and antioxidant pathways^28–33^. Our studies did not show activation of HIF-1α or upregulation of HIF target genes. While the lack of upregulation in our studies is consistent with the results from Polotsky et al.^28^, other studies have shown that IH activates HIF in ECs. The disagreement among studies raises questions about the differences in IH models employed by studies including differences in the severity of hypoxia (0–10% O2), duration of exposure to hypoxia, duration of IH (i.e., number of cycles) and the type of ECs used (arterial vs. venous). Some of the studies used intermittent anoxia, which induced the expression of proinflammatory cytokines at the levels similar to what was observed with continuous hypoxia^28,29^. The effect of IH was reported to depend on the duration of exposure to IH^29^. Studies in HUVECs showing IH-induced increased permeability and expression of adhesion molecules used longer duration of exposure to hypoxia (30–40 min) to model OSA^30,31^ limiting the relevance of findings to humans with OSA. Lastly, Kaczmarek et al. exposed human coronary artery EC and dermal microvascular ECs to IH (1% O_2_) and found that IH induced the gene expression of adhesion molecules and inflammatory cytokines as well as HIF-1α in coronary ECs, nut not in dermal microvascular ECs suggesting that the response to IH may be vascular bed dependent^33^. These findings do not agree with the other studies that showed that IH may directly induce EC activation^28–33^ likely due to the differences in IH models used. Collectively, further studies are needed to standardize IH models to best mimic the changes in O_2_ levels that occur in patients with OSA. Such an approach is necessary to improve our understanding about the mechanisms and will also allow comparison between different studies, which is not possible due to substantial differences in the models used at this time.

Interestingly, although epinephrine and IH alone regulated many genes similarly, when exposed simultaneously, the combinatory effect of IH on epinephrine-induced gene expression was inhibitory. In fact, IH had an opposite effect on the epinephrine-induced gene expression and attenuated the effects of epinephrine. As expected from these data, when we compared the gene expression under IH with IH and epinephrine together, there were only few genes that were differentially expressed consistent with loss of epinephrine-effect on gene expression. While further studies will be needed to understand the exact mechanisms behind these unexpected findings, we speculate that one possibility is that IH may exert beneficial effects on the cardiovascular system by activating antioxidant pathways^46,47^, which then may provide protection by inhibiting catecholamine-induced oxidative stress^48–50^. Contrary to the notion that OSA, which is characterized by IH as its hallmark, is an independent risk factor for cardiovascular disease, several studies have shown that IH conditioning may be cardioprotective both in experimental models as well as humans^51^. IH conditioning reduces infarct size and cardiac arrhythmias, and improves postischemia myocardial function. While the mechanisms are not completely understood, the duration and intensity of IH is thought to play a role in its cardioprotective effects as opposed to the unwanted effects attributed in OSA^29,51^. Consistent with our findings on the effects of IH on the epinephrine-induced transcriptional response, a recent study showed that IH exposure reduced markers of endothelial dysfunction in humans^52^. Furthermore, our findings corroborate data from clinical trials that compared supplemental oxygen, which treats IH, with CPAP therapy, which treats both IH and catecholamine release^53–55^. These trials failed to demonstrate cardiovascular benefit of supplemental oxygen (treatment of IH only) compared to CPAP, which remains the gold standard therapy emphasizing the importance of other pathophysiologic changes including catecholamines playing a bigger role than IH in the pathogenesis of cardiovascular disease. Further studies are needed to better understand the specific roles and complex interaction between IH and catecholamines not only on ECs, but also other cells within the cardiovascular system and other organs. Although the oxygen tension in our model was lower than those used for IH conditioning, it remains to be determined what the optimal oxygen concentration is to achieve IH conditioning. Future studies using a range of oxygen concentrations in IH models will be required to determine whether the inhibitory effect of IH on epinephrine-induced gene expression.

In our analysis, *MT1G* was identified as one of the few genes that is induced by combination of IH and epinephrine. MT1G encodes metallothionein, which is a metal binding protein involved in regulation of free levels of zinc^42^. Metallothionein has been shown to play an important role in oxidative stress, inflammation, and stress-related cell damage^42,56^. EC metallothionein expression has been shown to be regulated by blood flow pattern. ECs in parts of aorta where atherosclerosis is more likely to occur (i.e., aortic arch) are exposed to disturbed flow with low sheer stress. ECs from these atheroprone areas have higher expression of metallothionein compared to ECs in other parts of aorta where blood flow pattern is normal^57^. Exposure of ECs to disturbed flow in vitro also causes increased expression of metallothionein, which leads to increased binding of zinc and consequently decreases levels of free zinc promoting adhesion of monocytes and supporting a role for EC activation and dysfunction^57^. Interestingly, atherosclerotic areas have been shown to contain higher levels of zinc^58^. Our results suggest that metallothionein induced by IH and epinephrine may play a role in the pathogenesis of cardiovascular disease in OSA. Future experiments will be required to determine whether *MT1G or* metallothionein plays a causal role in the effects of IH on endothelial activation in vivo.

Our manuscript has several limitations. Our OSA model focused primarily on IH as the severity of hypoxia is associated with cardiovascular disease and did not include other pathophysiologic changes that occur in OSA including intermittent hypercapnia. Therefore, we cannot exclude the possibility of intermittent hypercapnia or other pathophysiologic changes that occur during an apnea having an effect on EC gene expression. As mentioned above, we only employed a single IH model in our experiments and did not study the impact of duration or the severity of hypoxia on EC function and gene expression. Furthermore, while the epinephrine dose used in our studies^34^ is within the dosing range used in in vitro studies^59–61^. It is possible that that different concentrations of epinephrine may have different effects and the observed effects on gene expression may be dose dependent.

In conclusion, we found that both IH and epinephrine induce changes in gene expression in ECs. The effect of epinephrine on gene expression is greater than IH alone in terms of number of genes and the changes in the level of expression. Addition of IH attenuated and reversed the epinephrine-induced changes in gene expression in ECs. Our studies also identified new pathways affected by IH and epinephrine. Further investigation of these pathways will increase understanding of the mechanisms by which OSA causes cardiovascular disease.

## Methods

### Cells

We used only primary human aortic endothelial cells (HAECs) in our studies. HAECs were purchased from Lonza (Allendale, NJ) (CC-2535, lot number 000035034 or 000035998). Cells were grown in EGM-2 supplemented with SingleQuots from Lonza (CC-3156 and CC-4176) and Penicillin/Streptomycin/Amphotericin B Solution (Sigma). Epinephrine hydrochloride (Sigma) was solubilized in water and kept in − 20 °C in aliquots of 10 mM of concentration. All primary cultures were used from passage 5–7.

### In vitro exposure of HAECs to intermittent hypoxia

For in vitro studies, HAECs were exposed to alternating cycles of 5% O_2_ for 2 min followed by 20% O_2_ for 5 min on a cyclic perfusion system with a flow rate of 5 ml/min. HAECs were exposed to either normoxia (control) or IH contemporaneously. Normoxia (control) cells were also exposed to flow like IH-exposed cells under the same perfusion system. There is no sheer stress due to perfusion system. Hypoxic and normoxic media were prepared by bubbling of appropriate gases prior to experiments. Both hypoxic and normoxic circuit have independent pumps ensuring intermittent, but continuous flow of the conditioned media on a 4-chamber interconnected culture dish. The whole system was operated at 37 °C. The CO_2_ level in the control normoxia and IH exposure was at 5% and balance was N_2_. O_2_ levels in the individual hypoxic and normoxic medium were monitored by a galvanic dissolved oxygen probe (Oakton, DO 6+).

### RNA isolation and sequencing

Total RNA was isolated from HAECs using, GenElute™ Mammalian Total RNA Miniprep Kit and submitted to the University of Chicago Genomics Core Facility for sequencing with the Illumina NovaSEQ6000 sequencer (100 bp paired-end). Sequencing read (FASTQ) files were generated and assessed for per base sequence quality using FastQC. RNA-seq reads were pseudoaligned using Kallisto v.0.44.0 the at University of Chicago, CRI Gardner high performance computing cluster^62^. The Kallisto index was made with default parameters and the GENCODE (Release 39 (GRCh38.p13) and was run in quant mode with default parameters. Following pseudoalignment, we computed gene abundances using R package tximport v.1.18.0^63^. Differential expression was calculated between normoxia and hypoxia groups using R package edgeR^64^. edgeR performs read count filtering, normalization, estimating dispersion, and identification of differentially expressed genes. Differential gene expression was considered significant for genes with an FDR-adjusted *p* value ≤ 0.05 and fold change (FC) > 2. An absolute fold change ≥ 2 and false discovery rate (FDR) adjusted *p* value ≤ 0.05 were used to select and classify the significant DEGs. The raw RNA-seq data are accessible via GEO (GSE205050). https://www.ncbi.nlm.nih.gov/geo/query/acc.cgi?acc=GSE205050.

All volcano plots were drawn using ggplot2 R package. All heatmaps were generated with Pretty heatmaps R package pheatmap package from Z-score normalized expression values. https://cran.r-project.org/web/packages/pheatmap/pheatmap.pdf Pathway enrichment analyses were performed, and plots were created using R clusterProfiler package^65^ or using Enrichr search engine web interface on Enrichr database^66^. All packages were run on RStudio (2021.09.0 Build 351) with R version 4.0.3. We also performed gene set enrichment analysis using KEGG data^67^.

### Quantitative PCR

RNA was isolated GenElute™ Mammalian Total RNA Miniprep Kit and reverse transcribed using iScript Reverse Transcription Supermix (Bio-Rad, catalog number 1708841). Quantitative mRNA expression was determined by real-time quantitative PCR (qPCR) using iTaq Universal SYBR Green Supermix (Bio-Rad, catalog number 172-5121). *RPL19 was* used as a housekeeping gene, and gene expression was quantified using the ΔΔct method to determine relative fold-change. Primer sequences were used in this study are:

- *CD34* (5′-CTTGCTGAGTTTGCTGCCTTC-3′, 5′-AGGTAACTCTGGGGTAGCAGT-3′)
- *EFNA1* (5′-ACAGTTCAAATCCCAAGTTCCG-3′, 5′-CGTCTGCCACAGAGTGATCT-3′)
- *FABP4* (5′-AACTGGTGGTGGAATGCGT-3′, 5′-GGTCAACGTCCCTTGGCTTA-3′)
- *IRF1* (5′-CAACTTCCAGGTGTCACCCA-3′, 5′-CGACTGCTCCAAGAGCTTCA-3′)
- *JUNB* (5′-CCACCTCCCGTTTACACCAA-3′, 5′-GAGGTAGCTGATGGTGGTCG-3′)
- *MT1G* (5′-GCCCTGCTCCCAAGTACAAA-3′, 5′-GCAAAGGGGTCAAGATTGTAGC-3′)
- *NR4A1* (5′-CAGGACCTGTTGCTGGAGTC-3′, 5′-GCCTGGCTTAGACCTGTACG-3′)
- *PLA2G4C* (5′-CAACACTCCCTTCCCACTCG-3′, 5′-TAGCCCGGATGGTCTCGAA-3′)
- *RPL19* (5′-AGTATGCTCAGGCTTCAGAAGA-3′, 5′-CATTGGTCTCATTGGGGTCTAAC-3′)
- *SELE* (5′-CCAAAAGGCTCCAATGTGGC-3′, 5′-GCATCGCATCTCACAGCTTC-3′)

#### Statistics

The qPCR data were analyzed in Prism 9 (GraphPad Software Inc., La Jolla, CA). All data are shown as mean ± standard deviation (SD). To determine significance, we used non-parametric tests including Mann–Whitney to compare two groups and Kruskal–Wallis test to analyze data with more than two groups requiring multiple comparisons. Statistical significance was defined as *p < 0.05.

## Supplementary Information

Supplementary Figures.

## Data Availability

The raw source data for RNA-seq are accessible via GEO (GSE205050). https://www.ncbi.nlm.nih.gov/geo/query/acc.cgi?acc=GSE205050.
