# Supplementary material for: Intermittent hypoxia inhibits epinephrine-induced transcriptional changes in human aortic endothelial cells
Source: Sci Rep. 2022 Oct 13;12:17167. doi: 10.1038/s41598-022-21614-5 (PMC9561121; doi:10.1038/s41598-022-21614-5)
Supplement: Supplementary file 1 — Supplementary Figures. [file 41598_2022_21614_MOESM1_ESM.pdf]

Supplementary Figure 1

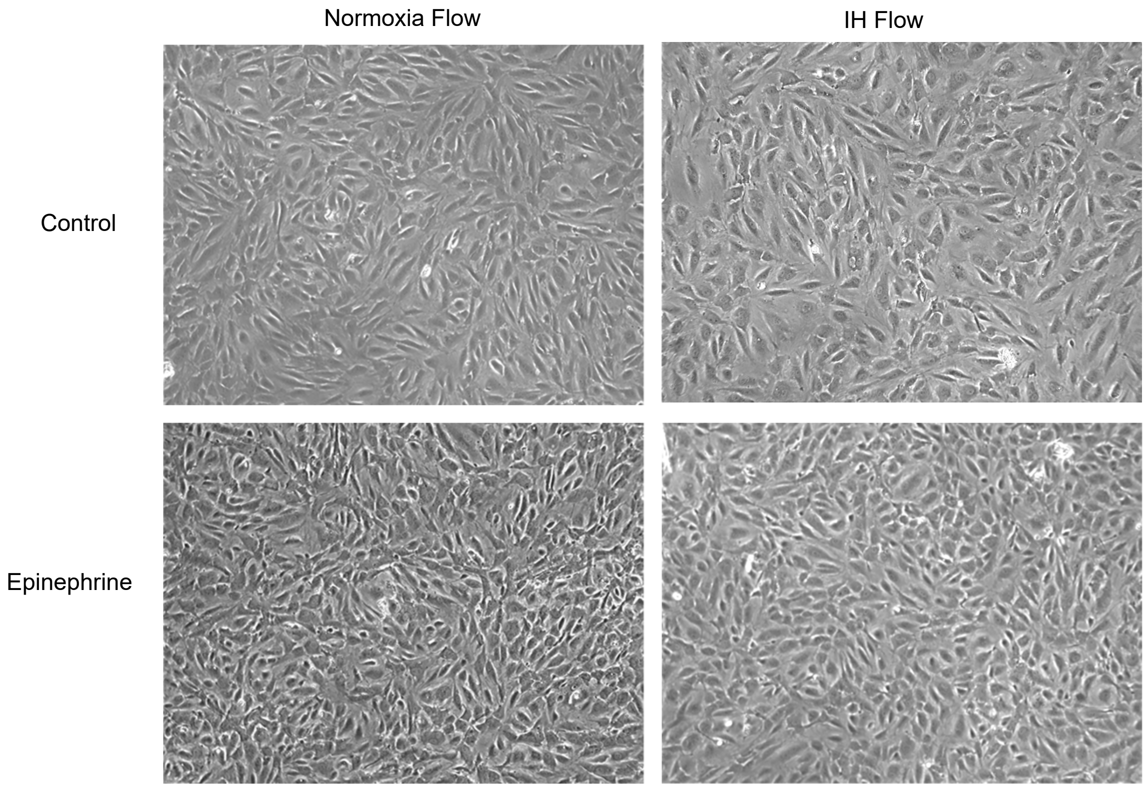

Supplementary Figure 1. Intermittent hypoxia does not cause any morphological changes in human aortic endothelial cells compared to control normoxia exposed cells. Images of human aortic endothelial cells were exposed to normoxia (20% O_2) or IH (5% O_2) for 60 cycles and treated with either control or epinephrine (10 μM).

Supplementary Figure 2

a

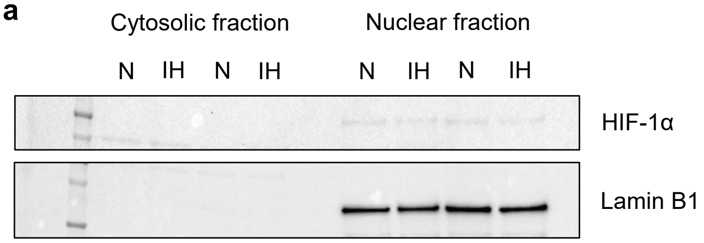

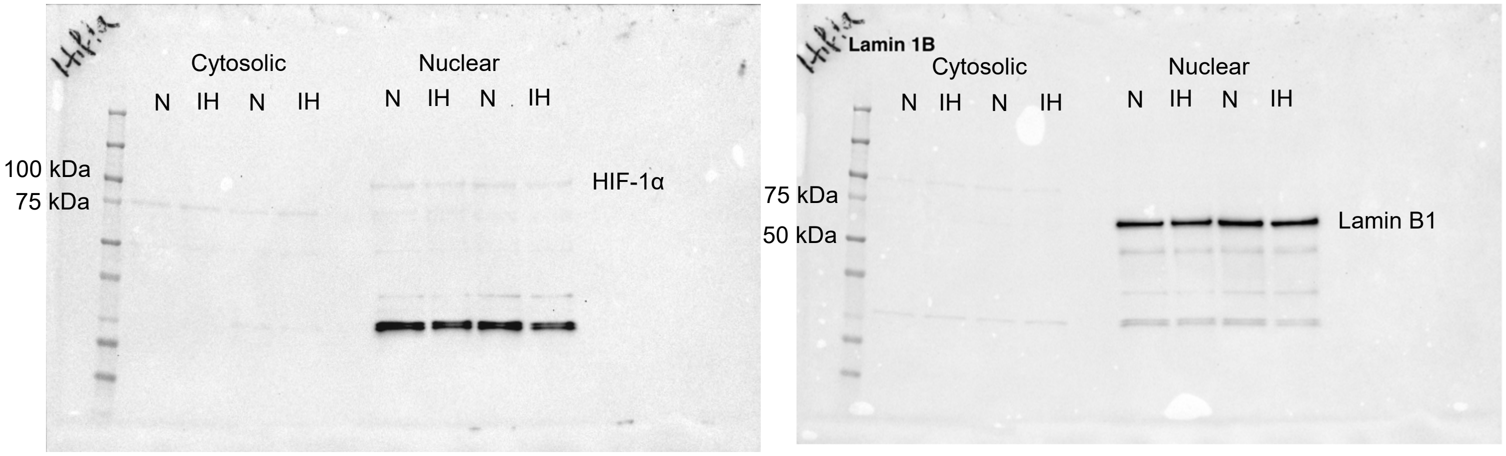

b

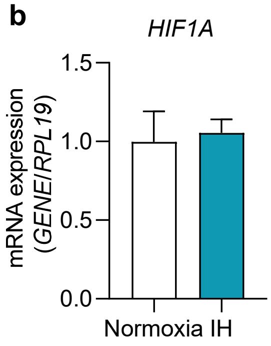

c

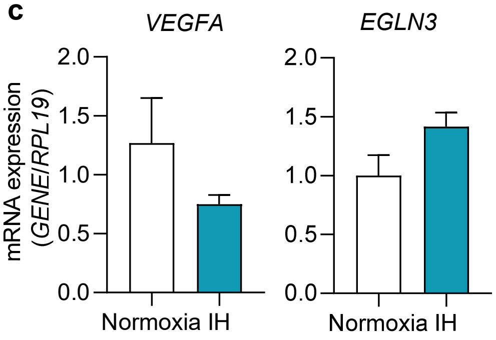

Supplementary Figure 2. Intermittent hypoxia does not induce HIF-1α or its target genes in human aortic endothelial cells. Human aortic endothelial cells were exposed to normoxia (N) (20% O2) or IH (5% O2) for 60 cycles. **(a)** HIF-1α protein expression was assessed by Western blot in cytosolic and nuclear fractions (full length, uncropped images were also provided) and **(b)** *HIF1A* gene expression and **(c)** HIF target gene (*VEGFA* and *EGLN3*) expression were assessed by qPCR**.**
